# Assessing professional behaviors: a self-administered scale for medical students during clerkships

**DOI:** 10.1186/s12909-024-05676-9

**Published:** 2024-06-26

**Authors:** Chunyu Xin, Xinzhi Song, Simeng Wang, Xuemei Cui, Ning Ding, Deliang Wen

**Affiliations:** Institute of Health Professions Education Assessment and Reform, China Medical University, No.77 Puhe Road, Shenyang North New Area, Shenyang, 110122 Liaoning Province China

**Keywords:** Scale development, Professional behavior, Medical professionalism, Medical student during clerkships, China

## Abstract

**Background:**

Medical professionalism is a core competency for medical students during clerkships for further professional development. Given that the behavior-based framework could provide clear insight and is easy to assess, the study aimed to create a self-administered scale to measure the professional behaviors of medical students during their clerkships.

**Methods:**

A comprehensive literature review on medical professional behaviors in English or Chinese and Delphi interviews were used to develop the initial version of the Self-Administered Scale for Professional Behavior of Medical Students During Clerkships. The reliability and validity analysis based on a survey of medical students from China, Cronbach’s α calculations, and Confirmatory Factor Analysis (CFA) specifically were conducted to finalize the scale. The associations of professional behaviors with gender, medical programs, and clerkship duration were examined using Wilcoxon rank-sum tests.

**Results:**

We included 121 studies and extracted 57 medical professionalism assessment tools, initially forming a pool of 48 items. To refine these items, eighteen experts participated in two rounds of Delphi interviews, ultimately narrowing down the item pool to 24 items. A total of 492 participants effectively completed the questionnaire. One item was removed due to its correlated item-total correlation (CITC) value, resulting in a final scale containing 23 items with six domains: *Respect, Altruism, Communication and Collaboration, Integrity, Duty, and Excellence.* The overall Cronbach’s alpha value was 0.98, ranging from 0.88 to 0.95 for each domain. The fit indices (χ^2^/df = 4.07, CFI = 0.96, TLI = 0.95, RMSEA = 0.08, and SRMR = 0.02) signified a good fit for the six-domain model. Medical students’ professional behavior was significantly associated with gender (*p* = 0.03) and clerkship duration (*p* = 0.001).

**Conclusion:**

The scale was demonstrated to be reliable and valid in assessing the professional behaviors of Chinese medical students during clerkships.

**Supplementary Information:**

The online version contains supplementary material available at 10.1186/s12909-024-05676-9.

## Introduction

In the patient-centric culture, healthcare was expected to focus on meeting the rising demands of patients, who now see themselves as *health service consumers* [1]. Patients increasingly seek high-quality care and upgraded service experiences [1]. This adds pressure on health professionals that patients not only require them to continuously improve their medical knowledge and skills to perform best practices [2] but also wish to be treated with compassion and friendliness [3]. However, with the growing commercialization of the healthcare system [4], economic rewards are often given priority over the pursuit of patients’ interests and well-being [5]. This has threatened medical professionalism, the contract between medicine and society [6]. Aiming to ease this tension, the Physician Charter was proposed in 2002, outlining three fundamental principles and ten professional responsibilities designed to guide medical professionals in their practice [7]. Medical professionalism, since then, has been recognized as the utmost responsibility of health professionals [8]. Empirical research has also reinforced the fact that without medical professionalism, clinical excellence cannot be achieved [9].

For medical students, future physicians, medical professionalism is a central competency to maintain and develop [10] to further their professional development [11]. Medical professionalism is a focus in undergraduate medical education [12]. However, medical students’ professionalism showed a downward trend, particularly during their clerkships [13, 14], possibly due to heavy workloads, the adverse impact of the hidden curriculum, or struggles in transitioning from classroom-based to patient-centered clinical learning [15–17]. This trend caused a significant concern because although students usually engage in inpatient or outpatient care under strict supervision during clerkships, their professional lapses could still occur and threaten healthcare outcomes ulteriorly. Moreover, professionalism lapses during undergraduate education profoundly impact future clinical performance in the workplace [18, 19]. Therefore, medical students’ professionalism in this phase needs more attention and actions from medical educators.

To provide a clear insight into medical professionalism, Irby and his colleagues have suggested three frameworks: virtue-based, behavior-based, and professional identity formation frameworks [20]. The behavior-based approach is the most commonly utilized and emphasized framework in literature, possibly because professional behaviors could reflect one’s professionalism status and are easy to assess and cultivate [21, 22]. As researchers suggested, with this approach, medical instructors could provide direct feedback to students, reinforce correct behaviors, and sanction unprofessional behaviors [20]. For example, a 360-degree evaluation tool was utilized to provide feedback on professional behavior from various stakeholders in the workplace [23]. Roff et al. developed a 41-item unprofessional behavior inventory and accordingly proposed appropriate sanctions, which is of great educational value [24].

Despite several scales being available [25], there are still two gaps in previous research. Firstly, most scales focus on participants’ understanding or attitude toward professionalism rather than their professional behaviors or clinical performance. However, the relationship between attitudes and behaviors is complicated and sometimes inconsistent [26], leading to significant measurement errors in professionalism status. Secondly, to the best of our knowledge, there are few, if any, scales specifically designed to assess medical students’ professional behaviors during their clerkships. During clerkships, like many countries and regions in the world, medical students in China are placed in real clinical settings, work as active medical team members, and participate in inpatient or outpatient care (i.e., observing patients, taking medical histories, conducting physical examinations, writing medical documents, managing patients, etc.) [27, 28]. Given the different and increasing requirements of professional behaviors during this training stage [20], a tool designed to assess medical students’ professional behaviors during their clerkships is warranted. Therefore, the study aimed to develop a self-administered assessment tool for the professional behaviors of medical students during their clerkships and validate it in Chinese medical students.

## Methods

The Self-Administered Scale for Professional Behavior of Medical Students During Clerkships was developed using a three-step process. Firstly, a literature review was conducted to create an item pool of professional behaviors. Then, the Delphi method was utilized to identify the significant items and establish face and content validity. Lastly, the scale was administered to Chinese medical students to examine its psychometric properties, including reliability and construct validity.

### Scale development

We conducted a broad literature review of existing literature on medical professional behavior assessment focusing on medical student samples using databases including PubMed, Web of Science, and CNKI. The search strings used in the study are shown in Additional file 1. Articles published between 2000 and 2022 in English or Chinese were selected.

After removing duplicates, 6029 articles were yielded, of which 2623 were in Chinese. C.X. and N.D. independently screened the title, abstract, and full article to select relevant articles. During the selection process, all authors discussed discrepancies until a consensus was reached. Altogether, 83 studies in the English language and 38 Chinese studies were included, yielding 57 medical professionalism assessment tools (see Additional file 2). We extracted the information containing instruments used, author/publication year, country, number of items, administration method, and domains of the instrument to describe further the articles included in the study.

All the survey items were collected from the included literature, and duplicated ones were omitted. C.X. and N.D. jointly merged similar items and modified the wording according to Chinese culture and customs, resulting in an item pool of 48 items. Since the study aimed to develop a behavior-based scale, each item contains two parts: an indicator and behavior descriptions. Regarding the items targeting the population of medical students during clerkships and reported instruments’ domains of previous studies, we categorized 48 items into six domains: *Respect*, *Altruism*, *Communication and Collaboration*, *Integrity*, *Duty*, and *Excellence*. Disagreements were resolved by plenary discussion.

A group of 18 experts from 11 institutions were contacted via e-mail for participation in the study. The expert panel inclusion criteria were as follows: (1) having a master’s degree or above; (2) engaging in clinical work, medical education, or medical education management for more than five years; and (3) holding the title of sub-senior or above. The experts who met all of the inclusion criteria were included in the panel. The experts rated 48 items from the initial pool according to their importance when evaluating medical students’ professional behavior. To screen out the weak items, a 5-point Likert scale was used for scoring, with the options ranging from *very unimportant* to *very important*. Their basis of judgment and degree of familiarity with medical professionalism were also evaluated. Furthermore, experts were asked to provide qualitative feedback on elusive, unnecessary, or redundant items for suggestions to modify, add, or remove. The consensus criteria of item retention were to meet all three criteria: (1) the mean value of importance was greater than 4 points, (2) more than 20% of the experts graded 5 points, and (3) the coefficient of variation (CV) was less than 0.25. The positive coefficient and the degree of authority were calculated.

### Survey administration

An online survey was implemented from June to July 2023 to screen the items of the pre-final scale and demonstrate the reliability and validity of the scale. We invited all students who enrolled in 2018 and 2019. At the time of the survey, students of 2018 were at the end of their clerkship, while students of 2019 were at the beginning instead. When recruiting participants, the research team introduced the importance of clerkship students behaving professionally and how the project could contribute towards enhancing the regulation of students’ professional behavior and supporting clinical teachers in remediating students’ unprofessional behaviors. Besides the project’s introduction, the online survey’s instructions clearly stated that the information collected was confidential and would only be used for research purposes. Therefore, the self-administered professional behavior scores based on the survey data would not affect participants’ clerkship grades. Participation in the survey was voluntary, and participants could quit at any time. And those who completed the questionnaire would receive a 20-CNY gift voucher worth about one cup of coffee in China.

The questionnaire included two parts: (1) personal information such as gender, clerkship duration (enrollment year in 2018 or 2019), and medical program (five- or eight-year); (2) a Self-Administered Scale for Professional Behavior of Medical Students During clerkships. The respondents’ behavioral frequencies were assessed using a five-point Likert scale, ranging from *never* to *always*, on a scale of 1 to 5.

### Analysis

Descriptive statistics were used to summarize individual characteristics. Item analysis was conducted to screen items using the correlation coefficient and Cronbach’s alpha coefficient methods. Those items with a correlated item-total correlation (CITC) greater than 0.50 were retained. Cronbach’s alpha coefficient was calculated to test internal consistency for the total scale and each domain, and values > 0.70 were considered acceptable [29, 30]. Any item that caused an increase in Cronbach’s alpha coefficient after its removal was eliminated.

Confirmatory factor analysis (CFA) was used to demonstrate the structure of the scale. Goodness-of-fit indices such as Chi-square and degrees of freedom ratio (χ^2^/df), Comparative Fit Index (CFI), Tucker Lewis Index (TLI), Root Mean Square Error of Approximation (RMSEA), and Standardized Root Mean Square Residual (SRMR) were used to assess the model fit. In theory, the model was deemed acceptable if χ^2^/df < 5.00 [31], CFI > 0.90, TLI > 0.90, RMSEA < 0.08, and SRMR < 0.08 [32]. Furthermore, Wilcoxon rank-sum tests were used to identify the differences in professionalism and each domain among different demographic characteristics.

All statistical analyses were performed using STATA (version 17.0; StataCorp, College Station, TX), and a two-tailed *p*-value < 0.05 was considered statistically significant.

### Ethics

The study protocol was approved by the China Medical University Ethics Committee (20231110145725). All expert panel members in the Delphi process were given sufficient explanations of the purpose of the study, and informed consent was obtained. The individual information was ensured to be kept anonymous and confidential. During the survey, to ensure anonymity, we did not collect information that could identify participants, such as names and IDs. The data gathered in this study are only used for research purposes and are not available to anyone outside the research team. As a result, the self-administered professional behavior scores cannot be linked to students’ clerkship grades. All the information above was given to the participants when obtaining their consent.

## Results

### Scale development

To refine the initial item pool, a two-round Delphi inquiry was conducted. The expert panel includes 11 males and seven females; five experts (27.78%) held senior titles. The positive coefficient was 100%, and the judgment coefficient, familiarity degree, and authority coefficient of the experts were 0.94, 0.78, and 0.86, respectively. Both quantitative and qualitative analyses of experts’ opinions were performed to modify, merge, or remove items.

According to the first round of the Delphi process, the mean value of the importance score ranged from 3.39 to 4.72, and 11 items were deleted (the importance value < 4.00, or proportion of 5 points < 20%, or CV > 0.25). Additionally, two items were removed based on experts’ suggestions. Considering the experts’ recommendations, three pairs of items were merged due to repetitive connotations, and five items were modified due to inappropriate or ambiguous expressions. Therefore, 32 items were formed after the first round of the Delphi process.

In the second round, the mean value of importance score ranged from 4.11 to 4.72. Eight redundant and unrelated items were removed according to experts’ qualitative suggestions. Three items were deleted because experts believed they were weakly related to the concept of professionalism, and three items were removed because of potentially lower discrimination in measurement. We also deleted two items due to their identical contents to other items. Also, three items were modified to be accurate expressions. Hence, the initial 24-item pool was created based on the selected items resulting from two rounds of Delphi methods, see Table 1.

**Table 1:** Scale for professional behavior of medical students during clerkships (English version)

Item	Indicator	Behavior descriptions
P1	Equality for every patient	Treat all patients with respect and equality, regardless of gender, age, culture, ethnicity, religion, sexual orientation, medical condition, or socioeconomic status.
P2	Respect for patients’ autonomy	Encourage patients and their families to participate in the decision-making process of diagnosis and treatment, provided that they are informed about the condition of the disease, treatment options, and the benefits and harms of each treatment option.
P3	Confidentiality of patients’ information	Avoid disclosing patients’ information or discussing patients’ privacy in public places and social media.
P4	Respect for patients’ body privacy	Consider patient’s psychological feelings and cover up to reduce the exposure of body parts of patients during physical examination or treatment.
P5	Respect for patients’ right to informed consent	Respect patients’ right to informed consent, and provide complete information about their condition, treatment plan, and potential risks involved in clinical diagnosis and treatment, clinical trials, or epidemiological investigations.
P6	Consideration of patients’ feelings	Take the initiative to care for patients, observe their emotions, and provide emotional and psychological support.
P7	Provision of convenience for patients	Fully consider patients’ rights and needs and schedule diagnosis and treatment services for them based on their convenience.
P8	Fair competition with others	Allow others to excel, and do not sacrifice others’ interests for your own advancement.
P9	Active listening to patient complaints	Encourage patients to share information about their condition and actively listen and respond appropriately.
P10	Patience in answering questions from patients	Answer the questions of patients clearly and patiently so that it is easy for them to understand.
P11	Gaining patients’ trust	Establish trust and cooperation with patients to avoid conflicts.
P12	Maintaining a positive medical interpersonal relationship	Show respect to other medical workers, establish good cooperative relationships, and avoid making derogatory comments or inappropriate statements about them on social media.
P13	Keeping honest and upright	Avoid seeking financial benefits or other undesirable temptations, including red envelopes, gifts, or any other forms of bribery.
P14	Strict adherence to medical practice guidelines	Strictly follow standard operating procedures, guidelines, and protocols in clinical practice, including medical document preparation, adherence to aseptic principles, etc.
P15	Avoidance of clinical activities exceeding capacities	Seek help from others when faced with problems beyond personal knowledge and skill levels while interacting with patients.
P16	Honest treatment of learning activities	Maintain credibility of testing and avoid plagiarism homework, and refrain from inviting or assisting classmates in signing up for academic lectures and conferences.
P17	Careful completion of clinical tasks	Do not avoid any clinical tasks that are within one’s ability, and make every effort to complete all assigned tasks.
P18	Organized execution of work	Organize and implement a clinical work plan and adjust as needed for unexpected situations.
P19	Commitment to schedule	Be punctual and do not miss any rounds, clinics, or other medical activities.
P20	Upholding professional reputation	Maintain a positive professional reputation and refrain from making inappropriate comments about doctors or the medical profession in front of patients in public or on social media.
P21	Awareness of weakness	Maintain a humble attitude, consistently reflect, acknowledge one’s limitations, accept criticism and feedback, and strive for self-improvement.
P22	Lifelong learning	Be aware of and enhance the ability for lifelong learning, pay attention to training opportunities and continue acquiring knowledge through various ways.
P23	Modesty for advice	Seek advice from others with an open mind when facing problems beyond one’s knowledge and abilities.
P24	Development of plans	Create clear long-term plans for both academic and career paths and actively work towards achieving those goals.

Note: Since the study administered the Chinese version of the scale, the English version needs to be further validated

Item P15 was dropped according to item analysis results in this study

### Scale validation and adjustment

Five hundred fifty-one students voluntarily participated, with 492 completing the questionnaires, yielding an effective response rate of 89.29%. The sample included 216 males, accounting for 43.90% of the total, closely reflecting the university’s male population proportion of 46.64%. Furthermore, 151 participants, making up 30.69% of the sample, were enrolled in an 8-year medical program (See Table 2). And 73.37% of the sample enrolled in 2019 and had just begun clerkships, a higher proportion compared to the clerkship students in the university. Specifically, 22.03% were in the 8-year medical program, and 50.00% were enrolled in 2019.

**Table 2:** Demographics of participants of this study

Variables	*n*(%)
Gender	
Male	216(43.90)
Female	276(56.10)
Medical program	
5-year	341(69.31)
8-year	151(30.69)
Enrollment year	
2019	361(73.37)
2018	131(26.23)

### Reliability

The correlations between each item and total score ranged from 0.40 to 0.90. According to the item exclusion criteria, item 15 in the domain *Integrity* was removed due to a CITC value of less than 0.50 and an increase in Cronbach’s alpha when deleted, see Table 3. As a result, the final version of the scale included 23 items, with 5, 3, 4, 3, 4, and 4 items distributed among the domains *Respect, Altruism, Communication and Collaboration, Integrity, Duty, and Excellence*, respectively.

**Table 3:** Correlated item-total correlation (CITC) for the scale for professional behavior of medical students during clerkship

Item	CITC value	Cronbach’s alpha value if item is deleted	Result
1	0.627	0.977	Keep
2	0.880	0.975	Keep
3	0.790	0.976	Keep
4	0.862	0.975	Keep
5	0.882	0.975	Keep
6	0.843	0.976	Keep
7	0.848	0.975	Keep
8	0.788	0.976	Keep
9	0.861	0.975	Keep
10	0.865	0.975	Keep
11	0.863	0.975	Keep
12	0.902	0.975	Keep
13	0.849	0.976	Keep
14	0.814	0.976	Keep
15	0.400	0.982	Drop
16	0.807	0.976	Keep
17	0.885	0.975	Keep
18	0.853	0.975	Keep
19	0.868	0.975	Keep
20	0.768	0.976	Keep
21	0.814	0.976	Keep
22	0.854	0.975	Keep
23	0.860	0.975	Keep
24	0.822	0.976	Keep

Descriptive data and Cronbach’s alpha value of the total scale and each domain are shown in Table 4. The total score ranged from 46 to 115, with a mean score of 106.21. The overall Cronbach’s alpha value was 0.98, ranging from 0.88 to 0.95 for each domain, indicating a high internal consistency reliability.

**Table 4:** Descriptive statistics of the scale for professional behavior of medical students during clerkships (*N* = 492)

Scale and domains	No. of items	Mean	SD	Min	Max	Cronbach’s alpha
Total scale	23	106.21	11.85	46	115	0.98
Respect	5	23.12	2.83	10	25	0.93
Altruism	3	13.83	1.64	6	15	0.91
Communication and Collaboration	4	18.53	2.11	8	20	0.95
Integrity	3	13.91	1.60	6	15	0.88
Duty	4	18.46	2.23	4	20	0.94
Excellence	4	18.36	2.35	4	20	0.95

### Validity

**Fig. 1:**
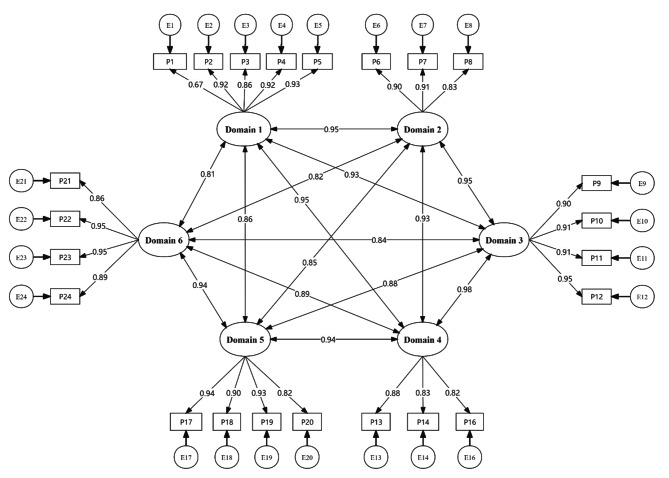
The internal structure of the six-domain model in chinese medical student. *Note*: Domain 1 - Respect; Domain 2 - Altruism; Domain 3 - Communication and Collaboration; Domain 4 – Integrity; Domain 5 – Duty; Domain 6 –Excellence

CFA was conducted to verify this study’s six-domain construct model’s structural fitting ability. Standardized factor loadings of all items and corresponding latent variables showed significant and high values ranging from 0.67 to 0.95, see Fig. 1. As indicated in Table 5, all fit indices were within the ideal range of standard criteria (χ^2^/df = 4.07, CFI = 0.96, TLI = 0.95, RMSEA = 0.08, and SRMR = 0.02). These results suggest that the scale performed well and that the six-domain construct model fits the Chinese medical student sample well.

**Table 5:** Goodness-fit indices for the six-domain model

Model	χ^2^/*n* (< 5.00)	CFI (> 0.90)	TLI (> 0.90)	RMSEA (< 0.08)	SRMR (< 0.08)
Six-domain model	4.07	0.96	0.95	0.08	0.02

### Connection with demographics

The differences in professional behavior scores between demographic groups are presented in Table 6. Females showed significantly better performance in medical professionalism than males (*p* = 0.03). This trend was also observed in *Respect, Altruism, Communication and Collaboration, Integrity*, and *Duty* domains. In contrast, no gender differences were found in the domain of *Excellence* (*p* = 0.07). Those nearly finishing clerkship training reported less frequent professional behavior corresponding to *Respect, Altruism, Communication and Collaboration, Integrity, and Excellence* domains than those who had just begun clerkships (*p* < 0.05). No significant differences in medical professional behavior frequencies regarding medical programs (5-year vs. 8-year) were found.

**Table 6:** Differences between individual characteristics(*N* = 492)

Variable	*n* (%)	Total Mean (SD)	Respect Mean (SD)	Altruism Mean (SD)	Communication and Collaboration Mean (SD)	Integrity Mean (SD)	Duty Mean (SD)	Excellence Mean (SD)
Gender								
Male	216(43.90)	104.37(13.46)	22.63(3.28)	13.53(1.90)	18.20(2.37)	13.67(1.79)	18.21(2.37)	18.13(2.51)
Female	276(56.10)	107.65(10.20)	23.50(2.36)	14.07(1.36)	18.78(1.86)	14.09(1.41)	18.65(2.11)	18.55(2.21)
Z value		-2.15	-2.74	-3.02	-2.79	-2.42	-2.07	-1.82
*P* value		0.03	0.006	0.003	0.005	0.02	0.04	0.07
Medical program								
5-year	341(69.31)	106.17(11.88)	23.14(2.88)	13.82(1.65)	18.51(2.11)	13.94(1.53)	18.43(2.28)	18.33(2.38)
8-year	151(30.69)	106.30(11.82)	23.09(2.73)	13.87(1.63)	18.57(2.12)	13.83(1.75)	18.52(2.13)	18.44(2.29)
Z value		0.23	0.35	-0.43	-0.50	0.38	-0.17	-0.33
*p* value		0.82	0.72	0.67	0.62	0.70	0.86	0.74
Enrollment year								
2019	361(73.37)	106.93(11.44)	23.32(2.72)	13.93(1.60)	18.62(2.06)	13.98(1.58)	18.58(2.08)	18.50(2.19)
2018	131(26.23)	104.23(12.73)	22.56(3.06)	13.56(1.72)	18.27(2.25)	13.70(1.65)	18.13(2.60)	18.00(2.35)
Z value		3.20	2.89	2.47	2.02	2.14	1.87	2.12
*p* value		0.001	0.004	0.01	0.04	0.03	0.06	0.03

## Discussion

We initially developed a scale to assess the professional behaviors of medical students during clerkships. The research involved a literature review, the Delphi method, and survey investigation, creating a reliable and valid scale containing 23 items under six domains: *Respect, Altruism, Communication and Collaboration, Integrity, Duty*, and *Excellence*. Our tool proved reliable and valid in evaluating professional behaviors among medical students during clerkships. Gender and clerkship duration were found to be associated with professional behaviors.

The scale developed in this study focused on professional behaviors instead of their understanding or attitude because such scales could mitigate two types of measurement errors of students’ professionalism status. One stems from the likelihood that students may struggle with professionalism challenges owing to the hidden curriculum in the workplace and fail to recognize their lapses [33]. When using scales of opinions, the error could be severe if the students lack a clear understanding of medical professionalism and appropriate professional behavior. The other is the widely-recognized gap between what medical students learn or think and what they do in practice [34]. The gap could originate from students’ incomplete transformation of knowledge into action, so even for those students with a proper understanding of professionalism, the gap could not be neglected. If ignoring these errors, merely depending on scales of understanding or attitude, clinical teachers could wrongly judge students’ actual status of professionalism. This could further undermine students’ transformation of knowledge into action and hinder realizing medical education’s purpose [34].

Although the self-administered scale developed in this study can also be used for observer-assessment, it was recommended that it be used for self-assessment. As discussed previously, some medical students may fail to recognize their lapses in professional behaviors owing to the hidden curriculum in the workplace [35]. However, all the scale’s items ask how frequently medical students’ certain behaviors occur, so even for those with no complete or accurate knowledge of professionalism, it would not be difficult to fill in the questionnaire [36]. Furthermore, regarding professional behavior assessment, self-administered scales have been proven reliable and valid in many certain professional behaviors during clerkships, such as the Pursuit of Excellence [37]. Additionally, the self-administered scale could offer a relatively quick and economical solution when collecting data from large-size samples. Whether self or other-rating, the scale could facilitate medical students to reflect on their clinical learning process and content, enabling them to regulate professional behaviors, and enhance their clinical performance [38, 39]. It could also help clinical teachers to early detect, constantly monitor, and timely remediate students’ unprofessional behaviors.

Medical professionalism is a culture-sensitive construct [40], and consequently, social or cultural variations could characterize preferences, aversions, and contrasts in professionalism assessments [10]. For example, as pointed out by one previous study, whether altruism is an integral element of medical professionalism was debatable across Asian and European cultures [41]. Hence, we conducted a literature review in English and Chinese languages in this study and hope to account for international perspectives and Chinese cultural background simultaneously, as well as balance cultural sensitivity and generalizability. The item generation in this study detected such cultural uniqueness and verified the rationale above. Protection for professional reputation and similar can only be found in Chinese literature. This finding might be related to the national health system reform in China launched about ten years ago, coping with the social contradictions caused by excessive marketization, such as the increase in out-of-pocket medical expenditure and the distrust between patients and physicians [42, 43]. Facing disputes between healthcare professionals and patients and widespread negative medical news, Chinese physicians and health authorities realized the need to redeem the reputation of healthcare professionals [43].

The study also confirms the rationality of the six-domain structure. There is no consolidated definition of medical professionalism worldwide [44], resulting in structural diversity in conceptual frameworks under different cultures [45]. Despite variations in terminology and expressions, the core connotation of the scale was consistent with existing professionalism frameworks. Five out of six domains of our scale (*Respect, Altruism, Integrity, Duty*, and *Excellence*) mirrored the professionalism construct proposed by American Board of Internal Medicine (ABIM), which was disseminated and used worldwide [7, 46]. The last independent domain in this study, *Communication and Collaboration*, aligns with previous studies verifying the importance of interpersonal relationships and teamwork [47, 48]. For example, Jha and his colleagues enlist *Communicating Effectively* in their framework of behavioral professionalism [49]. Including *Communication and Collaboration* acknowledges the critical role of teamwork and collaboration in healthcare outcomes [50]. After all, medical students are active medical team members and thus must master communication and collaboration competencies.

The preliminary testing results demonstrated that the Self-Administered Scale for Professional Behavior of Medical Students During clerkships is a psychometrically sound instrument to measure the frequencies of medical students’ professional behaviors during their clerkships. Through item analysis, one item was removed due to a low CITC value, resulting in the final scale with six domains and 23 items. Cronbach’s alpha of the total scale and each domain met the satisfactory criteria, indicating high homogeneity and internal consistency. The parameters of the model fitting degree of CFA were within an acceptable range, demonstrating good structural validity. We hope that the scale could be a valuable indicator for medical educators to evaluate medical students’ performance in their professional behaviors and identify those with unprofessional behaviors.

The study found that females scored significantly higher in professional behavior frequencies than males. This was consistent with former studies showing that females possess superior knowledge and attitudes toward medical professionalism than males [51, 52]. It was well recognized that females were more skilled in displaying professionalism-related behaviors, such as communication and empathy [14, 53], which were stereotypically associated with “female” characteristics [14]. Moreover, the study revealed that students at the end of their clerkships performed significantly worse in medical professional behavior than those just starting their clerkships. This was in line with the previous study that found a decline in medical students’ professionalism as their grades progressed [14, 54]. This decline might be attributed to gradual exposure to unprofessional and unethical behavior [14, 55] in the learning environment, and heavy workloads might also contribute to this trend [54].

There are several limitations in the study. Firstly, we conducted the investigation in a single university, which might restrict the generalizability of our findings. Secondly, the sample may suffer from an underrepresentation of 5-year program students and those enrolled in 2018 due to their potential struggles with preparing for the unified national graduate entrance examination while having clerkships [56, 57]. Thirdly, since the study was cross-sectional, test-retest reliability was not assessed in this study, and the trend in students’ professional behaviors during clerkship was not analyzed either. However, this work can serve as a foundation for administering the scale in diverse institutions for longitudinal surveys. Finally, since the scale was self-administered in this study, we cannot determine whether the students’ choices were influenced by perceived social desirability. Nonetheless, we could still detect differences in professional behaviors by gender and clerkship duration, demonstrating its effectiveness indirectly.

## Conclusions

A self-administered scale for measuring the professional behavior of medical students during their clerkships was developed by an extensive review of English and Chinese literature, followed by two rounds of Delphi methods and a survey investigation. The scale contained 23 items under six domains: Respect, *Altruism, Communication and Collaboration, Integrity, Duty*, and *Excellence*. Preliminary psychometric testing results showed good internal reliability and construct validity, indicating its reasonable use in Chinese medical students during clerkships.

### Electronic supplementary material

Below is the link to the electronic supplementary material.

Supplementary Material 1

Supplementary Material 2

## Data Availability

The datasets and Chinese version of the Self-Administered Scale for Professional Behavior of Medical Students During Clerkships used in the current study are available from the corresponding author, Deliang Wen, and Ning Ding upon reasonable request.
