# Supplementary material for: Assessing professional behaviors: a self-administered scale for medical students during clerkships
Source: BMC Med Educ. 2024 Jun 26;24:692. doi: 10.1186/s12909-024-05676-9 (PMC11200818; doi:10.1186/s12909-024-05676-9)
Supplement: Supplementary file 1 — Supplementary Material 1 [file 12909_2024_5676_MOESM1_ESM.docx]

**Additional file 1. Search strings used in the study**

**Web of Science**

TS=(Professionalism OR “Professional identity formation” OR “Professional attitude*” OR “Professional behavior*” OR “Professional performance*” OR “Professional misconduct*” OR “Unprofessional behavior*” OR “Professionalism lapse*” OR (Professional competenc*)) AND TS=(“Medical student*”) AND TS=(Scale* OR measure* OR inventor* OR questionnaire* OR tool* OR instrument* OR assess* OR evaluat* OR indicator* OR index* OR survey OR “self-report” OR test*)

**PubMed**

(“Professionalism”[Mesh] OR “Professionalism”[tiab] OR “Professional identity formation”[tiab] OR “Professional attitude*”[tiab] OR “Professional behavior*”[tiab] OR “Professional performance*”[tiab] OR “Professional misconduct*”[tiab] OR “Unprofessional behavior*”[tiab] OR “Professionalism lapse*”[tiab] OR (Professional competenc*)[tiab])) AND (“student, medical ”[Mesh] OR “Medical student*”[tiab]) AND (“Scale*”[tiab] OR “measure*”[tiab] OR “inventor*”[tiab] OR “questionnaire*”[tiab] OR “tool*”[tiab] OR “instrument*”[tiab] OR “assess*”[tiab] OR “evaluat*”[tiab] OR “indicator*”[tiab] OR “index*”[tiab] OR “survey”[tiab] OR “self-report”[tiab] OR “test*”[tiab])

**CNKI**

SU= (‘professionalism’+ ‘professional competency’ + ‘professional ethics’ + ‘professional identity formation’ + ‘professional identity’ + ‘professional attitude’ + ‘professional behavior’ + ‘competency’ ) * ‘medical student’

Note: The search string used in this study within the CNKI database was in Chinese language.
