# Supplementary material for: Assessing professional behaviors: a self-administered scale for medical students during clerkships
Source: BMC Med Educ. 2024 Jun 26;24:692. doi: 10.1186/s12909-024-05676-9 (PMC11200818; doi:10.1186/s12909-024-05676-9)
Supplement: Supplementary file 2 — Supplementary Material 2 [file 12909_2024_5676_MOESM2_ESM.docx]

Additional file 2. Literature included in the study

| **No.** | **Instrument** | **Authors/Year** | **Countries** | **Number of items** | **Administration** | **Instrument’s domain** |
| --- | --- | --- | --- | --- | --- | --- |
| 1 | PSCOM Professionalism Questionnaire | Blackall/ 2007[[1](#_ENREF_1)]; Akhund/ 2014[[2](#_ENREF_2)]; Bustamante/ 2014[[3](#_ENREF_3)]; Antes AL/ 2020[[4](#_ENREF_4)] | United States; Pakistan; Spain; China | 36 | Self-assessment | Development study: Accountability, Altruism, Duty, Excellence, Honesty and Integrity, and Respect |
| 2 | Tsai ABIM questionnaire | Tsai/ 2007[[5](#_ENREF_5)]; Yu FF/ 2019[[6](#_ENREF_6)]; Binh PDU/ 2021[[7](#_ENREF_7)]; Nhan/ 2014[[8](#_ENREF_8)]; Guo H/2022[[9](#_ENREF_9)] | China; Vietnam | China:32,23; Vietnam:23 | Self-assessment | Development study: Commitment to care; righteous and rule abiding; pursuing quality patient care; habit of professional practice; interpersonal relationship; patient-oriented’ issues; physician’s self-development; and respect for others  Validation study:   1. 6 domains: Self-Awareness, Professional Habit, Social Duty with, altruism, integrity, quality of care; 2. 4 domains: Honor and integrity, Practice habits and respect for others, Accountability and excellence, Duty |
| 3 | P-MEX | Cruess/ 2006[[10](#_ENREF_10)]; Parthiban N/ 2021[[11](#_ENREF_11)]; Liu J/ 2022[[12](#_ENREF_12)]; Karukivi M/ 2015[[13](#_ENREF_13)] | Canada; Malaysia; China; Finland | Canada, Malaysia, China:24; Finland:21 | Self-assessment and observer assessment | Development study: Doctor-patient relationship skills, reflective skills, time management, and interprofessional relationship skills |
| 4 | Dundee Polyprofessionalism Inventory I: Academic Integrity | Roff S/ 2011[[14](#_ENREF_14)]; Roff S/ 2012[[15](#_ENREF_15)]; Babelli S/ 2015[[16](#_ENREF_16)]; Sattar/ 2016[[17](#_ENREF_17)]; Sattar/ 2016[[18](#_ENREF_18)]; Sattar/ 2016[[19](#_ENREF_19)]; Guraya/ 2016[[20](#_ENREF_20)]; Guraya SY/ 2018[[21](#_ENREF_21)]; Sattar K/ 2021[[22](#_ENREF_22)]; Abdulrahman M/ 2017[[23](#_ENREF_23)]; Shukr I/ 2014[[24](#_ENREF_24)] | UK; Egypt; Saudi  Arabia; United Arab Emirates; Pakistan | UK:41,34; Egypt: 30; Saudi  Arabia:30,34; Arab Emirates:20; Pakistan: 47 | Self-assessment | None |
| 5 | Arnold’s Professionalism Climate Instrument (PCI) | Quaintance/ 2008[[25](#_ENREF_25)]; Spiwak R/ 2014[[26](#_ENREF_26)]; Hoobehfekr S/ 2021[[27](#_ENREF_27)]; Brazeau CM/ 2010[[28](#_ENREF_28)]; Al Gahtani HMS/ 2021[[29](#_ENREF_29)] | United States; Canada; Iran | United States:12,11; Iran:12; Canada:11 | Self-assessment,Students assess peers, residents, and faculties | Development study:none  Validation study:  (1) Respect/Caring/Compassion/Altruism; Honesty/integrity;  (2) Accountability/responsibility; Duty/Service/Excellence/Altruism |
| 6 | Medical Student Safety Attitudes and Professionalism Survey (MSSAPS) | Liao/ 2014[[30](#_ENREF_30)]; Keshmiri F/ 2022[[31](#_ENREF_31)]; Lee HY/ 2018[[32](#_ENREF_32)] | United States; Iran; Korea | 34 | Self-assessment | Development study: Safety culture, Teamwork culture, Error disclosure culture, Experiences with professionalism, Comfort expressing professional concerns |
| 7 | MacLeod Clark Professional Identity Scale | Toben, D/ 2021[[33](#_ENREF_33)]; Yu SR/ 2021[[34](#_ENREF_34)]; Chaou CH/ 2021[[35](#_ENREF_35)] | The Netherlands; China Taiwan | 10 | Self-assessment | None |
| 8 | Perceptions of professional misconduct survey | Hultman CS/ 2015[[36](#_ENREF_36)]; Hultman CS/ 2012[[37](#_ENREF_37)]; Wagner IJ/2013[[38](#_ENREF_38)]; Zulkifli J/ 2018[[39](#_ENREF_39)] | United States; Ireland | United States:30; Ireland:31 | Self-assessment | United States: breaches in honesty and integrity, lapses in confidentiality, boundary violations, conflict of interest, misuse of social media, self-prescribing, substance abuse, and discrimination;  Ireland:Inappropriate conduct not in relation to patient; Inappropriate non-sexual conduct towards patient; Illegal or unethical prescribing; Sexual relationship with patient; Supervision of others; Inappropriate or inadequate treatment; Breach of privacy; Failure to obtain informed consent; Academic; Research; Breach of conditions; Inappropriate use of social media; |
| 9 | Pritzker model of unprofessional behaviors checklist | Humphrey HJ/ 2007[[40](#_ENREF_40)]; Byszewski A/ 2012[[41](#_ENREF_41)]; Franco RS/ 2017[[42](#_ENREF_42)] | United States; Canada; Portugal | United States:12; Canada:17; Portugal:22 | Self-assessment | Development study:none;  Validation study:academic (A), clinic setting (C), and patient-related (P) |
| 10 | Professionalism attitude scale (PAS) | Ketis/ 2014[[43](#_ENREF_43)]; Selic P/ 2019[[44](#_ENREF_44)] | Slovenia | 22 | Self-assessment | Empathy and humanism,  Professional relationship and development, and Responsibility |
| 11 | Learner’s Attitude of Medical Professionalism Scale (LAMPS) | Eraky/ 2013[[45](#_ENREF_45)]; Rasul S/ 2021[[46](#_ENREF_46)] | Netherlands; Pakistan | 28 | Self-assessment | Duty/Accountability, Excellence/ Autonomy, Honor/ Integrity, Altruism, Respect |
| 12 | Jiang’s knowledge instrument | Jiang/ 2010[[47](#_ENREF_47)] | China | 24 | Self-assessment | Ethics, Skill, and Person |
| 13 | Cottrell’s peer professionalism assessment | Cottrell/ 2006[[48](#_ENREF_48)] | United States | 9 | Peer assessment | Honesty and Integrity, Accountability, Responsibility, Respectful and Nonjudgmental Behavior, Compassion and Empathy, Maturity, Skillful Communication, Confidentiality and Privacy in all patient affairs, Self-directed learning and appraisal skills |
| 14 | Medical Student Learning Competency | Bell H/ 2012[[49](#_ENREF_49)] | United States | 15 | Self-assessment | Regarding ethics  Regarding sensitivity and compassion  Regarding the ability to take responsibility |
| 15 | CanMEDS competency objectives | Rademakers JJ/ 2007[[50](#_ENREF_50)] | the  Netherlands | 4 | Self-assessment | Medical expert⁄ clinical decision maker, communicator, collaborator, manager, health advocate, scholar, professional |
| 16 | Mak-van's Unprofessional Behaviors inventory | Mak-van/ 2016[[51](#_ENREF_51)] | the  Netherlands | 109 | Observer assessment | 37 behavioral themes |
| 17 | Objective-centered rubric professionalism evaluation tool | Smith/ 2016[[52](#_ENREF_52)] | United States | 11 | Self-assessment | None |
| 18 | NTU professionalism MSF (NTU P-MSF) | Lee KL/ 2016[[53](#_ENREF_53)] | United States | 24 | Peer assessment and Self-assessment | Clinical knowledge and skills, communication skills, ethics, excellence, humanism, accountability, altruism, and integrity |
| 19 | Professionalism and ethics questionnaire | Yadav H/ 2019[[54](#_ENREF_54)] | Malaysia | 12 | Self-assessment | Discipline, plagiarism and cheating, and sexual harassment |
| 20 | Indiana University School of Medicine Self- and Peer Assessments Instrument | Hoffman LA/ 2017[[55](#_ENREF_55)] | United States | 18 | peer assessment and self-assessment | Work habits and interpersonal attributes |
| 21 | Vocational Identity Questionnaire | Leffel GM/ 2018[[56](#_ENREF_56)] | United States | 9 | Self-assessment | None |
| 22 | Tagawa's Professional identity formation scale | Tagawa M/ 2019[[57](#_ENREF_57)]; Tagawa M/ 2020[[58](#_ENREF_58)]; Findyartini A/2022[[59](#_ENREF_59)] | Japan; Indonesia | Japan; Indonesia27/15 | Self-assessment | Development study:self-control as a professional, awareness of being a medical doctor, reflection as a medical doctor, execution of social responsibility, and external and internal self-harmonization; SAS-2, SAS-3, SAS-4, and SAS-h of Kegan’s model;  Validation study:recognition and internalization of professional roles; self-control in professional behavior; reflections on professionalism; and thought processes as a medical/health professional |
| 23 | Scale of "Peer Nomination of Professionalism" | Mullikin TC/ 2019[[60](#_ENREF_60)] | United States | 10 | Peer assessment | Academic, Personal, and Future Potential as a Care Giver. |
| 24 | Students’ Inventory of Professionalism (SIP) | Noguera A/ 2019[[61](#_ENREF_61)] | Spain | 33 | Self-assessment | Holistic approach, caring for and understanding the patient, personal growth , teamwork, decision-making, patient assessment, and being a health care professional |
| 25 | Understandings of professionalism questionnaire | Salman Yousug Guraya/ 2021[[62](#_ENREF_62)] | Saudi Arabia, Malaysia, Abu dhabi | 22 | Self-assessment | None |
| 26 | Phase 2 Clinical Performance Evaluation - Surgery; Surgery CPE Form | Holmstrom AL/ 2021[[63](#_ENREF_63)] | United States | 8 | Observer assessment | None |
| 27 | Clerkship Evaluation Form | Gorth DJ/ 2021[[64](#_ENREF_64)] | United States | 23 | Observer assessment | None |
| 28 | Arnold’s Clerkship Professionalism Survey | Biagioli FE/ 2013[[65](#_ENREF_65)]; Sobani ZU/ 2013[[66](#_ENREF_66)]; Askarian M/ 2015[[67](#_ENREF_67)]; Ebrahimi S/ 2018[[68](#_ENREF_68)] | United States; Pakistan; Iran | United States, Pakistan:12; Iran:17 | Self-assessment | Excellence; Honor/integrity; Altruism/Respect |
| 29 | China Medical Student Survey (CMSS) Professionalism (PF) | Xu X/ 2022[[69](#_ENREF_69)]; Zhang G/ 2022[[70](#_ENREF_70)] | China | 10 | Self-assessment | None |
| 30 | Salam A's medical professionalism assessment scale | Haque M/ 2016[[71](#_ENREF_71)]; Kebede S/ 2018[[72](#_ENREF_72)] | Malaysia; Ethiopia | 44 | Self-assessment | Honesty, accountability, confidentiality, respectfulness, responsibility, compassion,  communication, maturity, and self-directed learning |
| 31 | CMSS Professional identity scale | Yu C/ 2022[[73](#_ENREF_73)] | China | 12 | Self-assessment | Professional cognition, professional emotion, professional behavior, and professional expectation |
| 32 | Englander’s common taxonomy scale | El Hage S/ 2022[[74](#_ENREF_74)] | Lebanon | 6 | Self-assessment | Patient care, knowledge for practice, practice-based learning and improvement, interpersonal and communication skills, professionalism, system-based practice, inter professional collaboration, and personal and professional development |
| 33 | Ten facets of competence scale | Bußenius L/ 2022[[75](#_ENREF_75)] | Germany | 10 | Self-assessment | Responsibility; Teamwork and collegiality; Empathy and openness; Knowing and maintaining own personal bounds and possibilities; Verbal communication with colleagues and supervisors; Structure, work planning and priorities; Empirically and scientifically grounded method of working; Active listening to patients; Coping with mistakes; Ethical awareness |
| 34 | Medical Professional Evaluation Scale (MPES) | Yamamoto T/ 2019[[76](#_ENREF_76)] | Japan | level 1:30; level 2:31 | Self-assessment | Level 1:Building interpersonal relationships, Planned learning, Interest in community health, Reflective practice, Knowledge and skills, Ethical and social  responsibility and Self-management; Level 2:Providing safe, quality care, Providing patient-centered care, Planned learning, Collaborative practice, Building interpersonal relationships, Interest in community health, Ethical and social responsibility and Reflective practice |
| 35 | EPMO | Imm MR/ 2022[[77](#_ENREF_77)] | United States | 5 | Observer assessment | None |
| 36 | Professional attitude scale | Yavari N/ 2021[[78](#_ENREF_78)] | Iran | 17 | Self-assessment | Patient-Centered Care; Prioritization of Patients’ Interests; Honesty; Accountability; Dealing with Misconducts |
| 37 | Professionalism, communication and collaboration scale | Ramakrishna J/ 2014[[79](#_ENREF_79)] | Canada | 10 | Observer assessment, peer assessment and self-assessment | None |
| 38 | Digital Professionalism scale | Mostaghimi A/ 2017[[80](#_ENREF_80)] | United States | 35 | Self-assessment | Privacy, information security, communications, and social media, boundaries, and online tone |
| 39 | Unprofessional behavior inventory | Jamalabadi Z/ 2018[[81](#_ENREF_81)] | Iran | 29 | Self-assessment | none |
| 40 | Communication/professionalism form (patient version) | Davis LE/ 2012[[82](#_ENREF_82)] | United States | 5 | Patients assess students | None |
| 41 | Chisholm’s professionalism assessment tool | AlKhater SA/ 2021[[83](#_ENREF_83)] | Saudi Arabia | 18 | Self-assessment | Excellence, Respect for Others, Altruism, Duty, Accountability, Honour and Integrity |
| 42 | Professional and unprofessional behaviors inventory | Tabatabaei ZS/ 2022[[84](#_ENREF_84)] | Iran | Professional 59; unprofessional 49 | Self-assessment | Altruism Honor and Integrity Responsibility/ Conscientiousness Respect Justice Excellence |
| 43 | medical interns’  professionalism evaluation form”of CMB project committee | Jiang S/2015[[85](#_ENREF_85)]; Shi Y/2019[[86](#_ENREF_86)]; Li Y /2015[[87](#_ENREF_87)]; Liang J/ 2015[[88](#_ENREF_88)]; Zhang W/ 2021[[89](#_ENREF_89)]; Sun C/2018[[90](#_ENREF_90)]; Li R/2016[[91](#_ENREF_91)] | China | 29 | Self-assessment/ observer assessment | Altruism, honor and integrity, care and communication, respect, responsibility, pursuit of excellence, leadership |
| 44 | Zhang’s Medical students’ professional identity scale | Zhang S/2009[[92](#_ENREF_92)]; Zhang L /2016[[93](#_ENREF_93)] | China | 24 | Self-assessment | Emotional recognition, ability recognition, professional will, professional expectations, professional values, professional recognition |
| 45 | Medical Ethics Standards and Implementation Measures for Medical Personnel | Zhao X/2009[[94](#_ENREF_94)]; Gao M/2003[[95](#_ENREF_95)] | China | 7; 8 | Self-assessment | None |
| 46 | Medical students’ professionalism cognition scale | Cai X / 2020[[96](#_ENREF_96)]; Sun J /2018[[97](#_ENREF_97)]; Yuan Y / 2022[[98](#_ENREF_98)] | China | 25; 27; 21 | Self-assessment | Profession cognition, profession emotion, profession will |
| 47 | Sun’s medical students’ professionalism cognition scale | Sun M/ 2021[[99](#_ENREF_99)] | China | 25 | Self-assessment | Academic competence, communication and practice, respect and care, responsibility, self-development |
| 48 | Liu’s medical professional identity scale | Liu W/2019[[100](#_ENREF_100)] | China | 22 | Self-assessment | Vocational cognition, vocational commitment, vocational behavior, vocational emotion, vocational expectation and vocational values |
| 49 | Professional identity and professionalism questionnaire | Deng Y/2020[[101](#_ENREF_101)] | China | Professional identity:11; medical professionalism:25 | Self-assessment | Professional identity: professional cognition, emotion, and matching; Medical professionalism: patient first, benevolence, equality, honesty, integrity, confidentiality, respect, peer treating, social responsibility, ideal and belief, lifelong learning |
| 50 | Perceptions on medical professionalism scale | Fu Y/2016[[102](#_ENREF_102)] | China | 14 | Self-assessment | None |
| 51 | Index System of Medical Students’ Professionalism Evaluation | Li Y/2020[[103](#_ENREF_103)] | China | 27 | Self-assessment | Professional attitude; Professional ethics; Professional awareness; Professional style |
| 52 | Tracking Evaluation Index System of Clinical Medicine Graduates' Training Quality | Xu Y/2020[[104](#_ENREF_104)] | China | 5 | Self-assessment | Humanistic, ethics, empathy, teamwork, responsibility |
| 53 | Evaluation index system of post competence of  clinical medical undergraduates | Chen M/2018[[105](#_ENREF_105)] | China | 13 | Observer assessment | Professional values; work adaptability; critical thinking ability |
| 54 | Chinese milestone standards for cultivating medical professionalism | Song W/2020[[106](#_ENREF_106)] | China | 21 | Self-assessment | Compassion, respect, communication and collaboration; Persuade excellent; Integrity, honest, and responsibility; The equitable distribution of medical resources, humanitarianism, and ethical standards |
| 55 | Clinical medical students’ professional identity scale | Wang Y/2015[[107](#_ENREF_107)] | China | 38 | Self-assessment | Motive, cognition, support, affection, anticipation, will |
| 56 | Medical students' e-professionalism behavior scale | Lv J/2020[[108](#_ENREF_108)] | China | 10 | Self-assessment | None |
| 57 | Medical students’ professional identity questionnaire | Zhang L/2010[[109](#_ENREF_109)]; Wu Y /2022[[110](#_ENREF_110)]; Qiu S/2022[[111](#_ENREF_111)]; Hao J/2021[[112](#_ENREF_112)]; Guo Z/2018[[113](#_ENREF_113)]; Lv Y/2020[[114](#_ENREF_114)]; Zhu K/2021[[115](#_ENREF_115)]; Pan L /2021[[116](#_ENREF_116)]; Cao X /2017[[117](#_ENREF_117)]; Wang F /2018[[118](#_ENREF_118)]; Mei S /2019[[119](#_ENREF_119)]; Yang Q /2018[[120](#_ENREF_120)]; Wu J /2014[[121](#_ENREF_121)] | China; China and America | China:38; China and America:31 | Self-assessment | Vocational cognition, vocational commitment, vocational behavior, vocational emotion, vocational expectation and vocational values |

**References**

1. Blackall GF, Melnick SA, Shoop GH, George J, Lerner SM, Wilson PK, Pees RC, Kreher M. Professionalism in medical education: the development and validation of a survey instrument to assess attitudes toward professionalism. Med Teach. 2007;29(2-3):e58-e62.

2. Akhund S, Shaikh ZA, Ali SA. Attitudes of Pakistani and Pakistani heritage medical students regarding professionalism at a medical college in Karachi, Pakistan. BMC Res Notes. 2014;7:150.

3. Bustamante E, Sanabria Á. Spanish adaptation of The Penn State College of Medicine Scale to assess professionalism in medical students. Biomedica. 2014;34(2):291-99.

4. Antes AL, Dineen KK, Bakanas E, Zahrli T, Keune JD, Schuelke MJ, DuBois JM. Professional decision-making in medicine: Development of a new measure and preliminary evidence of validity. PloS One. 2020;15(2):e0228450.

5. Tsai T-C, Lin C-H, Harasym PH, Violato C. Students' perception on medical professionalism: the psychometric perspective. Med Teach. 2007;29(2-3):128-34.

6. Yu F-F, Du C-Y, Liu Z-F, Chen L-J, Huang Y-X, Zhang L-L. Development and Validation of a Simplified Chinese Version of the Assessment Tool for Students' Perceptions of Medical Professionalism. Curr Med Sci. 2019;39(4):670-78.

7. Binh PDU, An PL, Nguyen NA, Nguyen DV, Huynh G, Gomi H, Yoshida M. Health Science Students' Perspective on Quality-of-Care-Relating Medical Professionalism. J Multidiscip Healthc. 2021;14:2229-38.

8. Nhan VT, Violato C, Le An P, Beran TN. Cross-cultural construct validity study of professionalism of Vietnamese medical students. Teach Learn Med. 2014;26(1):72-80.

9. Guo H, Guo R, Zhao Y, Liu H, Li X. Study on medical student's perceptions and influencing factors related to the development of medical professionalism. Chin J Med Educ, 2022:314-17 (in Chinese).

10. Cruess R, McIlroy JH, Cruess S, Ginsburg S, Steinert Y. The Professionalism Mini-evaluation Exercise: a preliminary investigation. Acad Med. 2006;81(10 Suppl):S74-S78.

11. Parthiban N, Boland F, Fadil Azim DH, Pawlikowska T, O'Shea MT, Jaafar MH, Morgan K. Asian medical students' attitudes towards professionalism. Med Educ Online. 2021;26(1):1927466.

12. Liu J, Jiao X, Zeng S, Li H, Jin P, Chi J, Liu X, Yu Y, Ma G, Zhao Y, Li M, Peng Z, Huo Y, Gao Q-L. Oncological big data platforms for promoting digital competencies and professionalism in Chinese medical students: a cross-sectional study. BMJ Open. 2022;12(9):e061015.

13. Karukivi M, Kortekangas-Savolainen O, Saxén U, Haapasalo-Pesu K-M. Professionalism Mini-Evaluation Exercise in Finland: A preliminary investigation introducing the Finnish version of the P-MEX instrument. J Adv Med Educ Prof. 2015;3(4):154-58.

14. Roff S, Chandratilake M, McAleer S, Gibson J. Preliminary benchmarking of appropriate sanctions for lapses in undergraduate professionalism in the health professions. Med Teach. 2011;33(3):234-38.

15. Roff S, Chandratilake M, McAleer S, Gibson J. Medical student rankings of proposed sanction for unprofessional behaviours relating to academic integrity: results from a Scottish medical school. Scott Med J. 2012;57(2):76-79.

16. Babelli S, Chandratilake M, Roff S. Egyptian medical students' recommended responses to the Dundee Polyprofessionalism Inventory I: Academic Integrity. Med Teach. 2015;37(3):277-80.

17. Sattar K, Roff S, Meo SA. Similarities and variances in perception of professionalism among Saudi and Egyptian Medical Students. Pak J Med Sci. 2016;32(6):1390-95.

18. Sattar K, Roff S. Comparison of recommended sanctions for lapses in professionalism of undergraduate medical students in a Saudi Arabian and a Scottish medical school. Med Teach. 2016;38(12):1262-66.

19. Sattar K, Roff S, Meo SA. Your professionalism is not my professionalism: congruence and variance in the views of medical students and faculty about professionalism. BMC Med Educ. 2016;16(1):285.

20. Guraya SY, Norman RI, Roff S. Exploring the climates of undergraduate professionalism in a Saudi and a UK medical school. Med Teach. 2016;38(6):630-32.

21. Guraya SY. Comparing recommended sanctions for lapses of academic integrity as measured by Dundee Polyprofessionalism Inventory I: Academic integrity from a Saudi and a UK medical school. J Chin Med Assoc. 2018;81(9):787-95.

22. Sattar K, Akram A, Ahmad T, Bashir U. Professionalism development of undergraduate medical students: Effect of time and transition. Medicine (Baltimore). 2021;100(9):e23580.

23. Abdulrahman M, Alsalehi S, Husain ZSM, Nair SC, Carrick FR. Professionalism among multicultural medical students in the United Arab Emirates. Med Educ Online. 2017;22(1):1372669.

24. Shukr I. Adaptation and validation of Dundee Polyprofessionalism inventory-1 for use in Pakistan. J Ayub Med Coll Abbottabad. 2014;26(4):548-53.

25. Quaintance JL, Arnold L, Thompson GS. Development of an instrument to measure the climate of professionalism in a clinical teaching environment. Acad Med. 2008;83(10 Suppl):S5-S8.

26. Spiwak R, Mullins M, Isaak C, Barakat S, Chateau D, Sareen J. Medical students' and postgraduate residents' observations of professionalism. Educ Health (Abingdon). 2014;27(2):193-99.

27. Hoobehfekr S, Asghari F, Sayarifard A, Kadivar M, Kashefinejad S. Medical students' perception of professionalism climate in clinical settings. J Med Ethics Hist Med. 2021;14:10.

28. Brazeau CMLR, Schroeder R, Rovi S, Boyd L. Relationships between medical student burnout, empathy, and professionalism climate. Acad Med. 2010;85(10 Suppl):S33-S36.

29. Al Gahtani HMS, Jahrami HA, Silverman HJ. Perceptions of medical students towards the practice of professionalism at the Arabian Gulf University. BMC Med Educ. 2021;21(1):38.

30. Liao JM, Etchegaray JM, Williams ST, Berger DH, Bell SK, Thomas EJ. Assessing medical students' perceptions of patient safety: the medical student safety attitudes and professionalism survey. Acad Med. 2014;89(2):343-51.

31. Keshmiri F, Raadabadi M. Assessment of safety attitudes, professionalism and exploration of medical students' experiences. BMC Med Educ. 2022;22(1):321.

32. Lee H-Y, Hahm M-I, Lee SG. Undergraduate medical students' perceptions and intentions regarding patient safety during clinical clerkship. BMC Med Educ. 2018;18(1):66.

33. Toben D, Mak-van der Vossen M, Wouters A, Kusurkar RA. Validation of the professional identity questionnaire among medical students. BMC Med Educ. 2021;21(1):359.

34. Yu S-R, Cheng Y-C, Tseng H-M, Chang Y-C, Ma S-D, Huang C-D, Hsieh M-J, Fang J-T, Chaou C-H. Undergraduates' preparedness for practice is associated with professional identity and perception of educational environment: A validation study. Biomed J. 2021;44(4):495-503.

35. Chaou C-H, Yu S-R, Chang Y-C, Ma S-D, Tseng H-M, Hsieh M-J, Fang J-T. The evolution of medical students' preparedness for clinical practice during the transition of graduation: a longitudinal study from the undergraduate to postgraduate periods. BMC Med Educ. 2021;21(1):260.

36. Hultman CS, Wagner IJ. Professionalism in plastic surgery: attitudes, knowledge, and behaviors in medical students compared to surgeons in training and practice--one, but not the same. Ann Plast Surg. 2015;74 Suppl 4:S247-S54.

37. Hultman CS, Connolly A, Halvorson EG, Rowland P, Meyers MO, Mayer DC, Drake AF, Sheldon GF, Meyer AA. Get on your boots: preparing fourth-year medical students for a career in surgery, using a focused curriculum to teach the competency of professionalism. J Surg Res. 2012;177(2):217-23.

38. Wagner IJ, Hultman CS. Elevation: developing a mentorship model to raise the next generation of plastic surgery professionals. Ann Plast Surg. 2013;70(5):606-12.

39. Zulkifli J, Noel B, Bennett D, O'Flynn S, O'Tuathaigh C. Medical students' perceptions of professional misconduct: relationship with typology and year of programme. J Med Ethics. 2018;44(2):133-37.

40. Humphrey HJ, Smith K, Reddy S, Scott D, Madara JL, Arora VM. Promoting an environment of professionalism: the University of Chicago "Roadmap". Acad Med. 2007;82(11):1098-107.

41. Byszewski A, Hendelman W, McGuinty C, Moineau G. Wanted: role models--medical students' perceptions of professionalism. BMC Med Educ. 2012;12:115.

42. Franco RS, Franco CAG, Kusma SZ, Severo M, Ferreira MA. To participate or not participate in unprofessional behavior - Is that the question? Med Teach. 2017;39(2):212-19.

43. Klemenc-Ketis Z, Vrecko H. Development and validation of a professionalism assessment scale for medical students. Int J Med Educ. 2014;5:205-11.

44. Selic P, Cerne A, Klemenc-Ketis Z, Petek D, Svab I. Attitudes toward professionalism in medical students and its associations with personal characteristics and values: a national multicentre study from Slovenia raising the question of the need to rethink professionalism. Adv Med Educ Pract. 2019;10:437-46.

45. Al-Eraky MM, Chandratilake M, Wajid G, Donkers J, van Merrienboer J. Medical professionalism: development and validation of the Arabian LAMPS. Med Teach. 2013;35 Suppl 1:S56-S62.

46. Rasul S, Zahid Bashir M, Saleem S, Tahir S, Rasheed A, Ali Sabir M. Assessment of Medical Professionalism among Students and Faculty Members of Shalamar Medical and Dental College, Lahore. J Adv Med Educ Prof. 2021;9(4):204-10.

47. Jiang S, Yan Z, Xie X, Tang W, Lu F, He J. Initial knowledge of medical professionalism among Chinese medical students. Med Teach. 2010;32(12):961-70.

48. Cottrell S, Diaz S, Cather A, Shumway J. Assessing Medical Student Professionalism: An Analysis of a Peer Assessment. Med Educ Online. 2006;11(1):4587.

49. Bell H, Donohue W, Liang YJ, Kim J, Cettin M. Osteopathic medical student learning competency. Fam Med. 2012;44(3):194-201.

50. Rademakers JJDJM, de Rooy N, Ten Cate OTJ. Senior medical students' appraisal of CanMEDS competencies. Med Educ. 2007;41(10):990-94.

51. Mak-van der Vossen MC, van Mook WNKA, Kors JM, van Wieringen WN, Peerdeman SM, Croiset G, Kusurkar RA. Distinguishing Three Unprofessional Behavior Profiles of Medical Students Using Latent Class Analysis. Acad Med. 2016;91(9):1276-83.

52. Smith KM, Geletta S, McArdle A. The Use of Rubrics in the Clinical Evaluation of Podiatric Medical Students: Objectification of the Subjective Experience. J Am Podiatr Med Assoc. 2016;106(1):60-67.

53. Lee K-L, Tsai S-L, Chiu Y-T, Ho M-J. Can student self-ratings be compared with peer ratings? A study of measurement invariance of multisource feedback. Adv Health Sci Educ Theory Pract. 2016;21(2):401-13.

54. Yadav H, Jegasothy R, Ramakrishnappa S, Mohanraj J, Senan P. Unethical behavior and professionalism among medical students in a private medical university in Malaysia. BMC Med Educ. 2019;19(1):218.

55. Hoffman LA, Shew RL, Vu TR, Brokaw JJ, Frankel RM. The Association Between Peer and Self-Assessments and Professionalism Lapses Among Medical Students. Evalu Health Prof. 2017;40(2):219-43.

56. Leffel GM, Oakes Mueller RA, Ham SA, Karches KE, Curlin FA, Yoon JD. Project on the Good Physician: Further Evidence for the Validity of a Moral Intuitionist Model of Virtuous Caring. Teach Learn Med. 2018;30(3):303-16.

57. Tagawa M. Development of a scale to evaluate medical professional identity formation. BMC Med Educ. 2019;19(1):63.

58. Tagawa M. Scales to evaluate developmental stage and professional identity formation in medical students, residents, and experienced doctors. BMC Med Educ. 2020;20(1):40.

59. Findyartini A, Greviana N, Felaza E, Faruqi M, Zahratul Afifah T, Auliya Firdausy M. Professional identity formation of medical students: A mixed-methods study in a hierarchical and collectivist culture. BMC Med Educ. 2022;22(1):443.

60. Mullikin TC, Shahi V, Grbic D, Pawlina W, Hafferty FW. First Year Medical Student Peer Nominations of Professionalism: A Methodological Detective Story about Making Sense of Non-Sense. Anat Sci Educ. 2019;12(1):20-31.

61. Noguera A, Arantzamendi M, López-Fidalgo J, Gea A, Acitores A, Arbea L, Centeno C. Student's Inventory of Professionalism (SIP): A Tool to Assess Attitudes towards Professional Development Based on Palliative Care Undergraduate Education. Int J Environ Res Public Health. 2019;16(24).

62. Guraya SY, Sulaiman N, Guraya SS, Yusoff MSB, Roslan NS, Al Fahim M, Abdelrehman D, Khoshhal S. Understanding the climate of medical professionalism among university students; A multi-center study. Innov Educ Teach Int. 2021;58(3):351-60.

63. Holmstrom AL, Chia MC, O'Brien CL, Odell DD, Burke J, Halverson AL. Entrustable Professional Activity-Based Summative Performance Assessment in the Surgery Clerkship. J Surg Educ. 2021;78(4):1144-50.

64. Gorth DJ, Magee RG, Rosenberg SE, Mingioni N. Gender Disparity in Evaluation of Internal Medicine Clerkship Performance. JAMA Netw Open. 2021;4(7):e2115661.

65. Biagioli FE, Rdesinski RE, Elliot DL, Chappelle KG, Kwong KL, Toffler WL. Surgery clerkship evaluations drive improved professionalism. J Surg Educ. 2013;70(1):149-55.

66. Sobani Z-u-a, Mohyuddin MM, Farooq F, Qaiser KN, Gani F, Bham NS, Raheem A, Mehraj V, Saeed SA, Sharif H, Sheerani M, Zuberi RW, Beg MA. Professionalism in medical students at a private medical college in Karachi, Pakistan. J Pak Med Assoc. 2013;63(7):935-39.

67. Askarian M, Ebrahimi Nia MJ, Sadeghipur F, Danaei M, Momeni M. Shiraz medical students' perceptions of their colleagues' professional behavior. J Adv Med Educ Prof. 2015;3(3):111-16.

68. Ebrahimi S, Atazadeh F. Medical Students' Occupational Burnout and its Relationship with Professionalism. J Adv Med Educ Prof. 2018;6(4):162-67.

69. Xu X, Bos NA, Wu H. The relationship between medical student engagement in the provision of the school's education programme and learning outcomes. Med Teach. 2022;44(8):900-06.

70. Zhang G, Wu H, Xie AN, Cheng H. The association between medical student research engagement with learning outcomes. Med Educ Online. 2022;27(1):2100039.

71. Haque M, Zulkifli Z, Haque SZ, Kamal ZM, Salam A, Bhagat V, Alattraqchi AG, Rahman NIA. Professionalism perspectives among medical students of a novel medical graduate school in Malaysia. Adv Med Educ Pract. 2016;7:407-22.

72. Kebede S, Gebremeskel B, Yekoye A, Menlkalew Z, Asrat M, Medhanyie AA. Medical professionalism: perspectives of medical students and residents at Ayder Comprehensive and Specialized Hospital, Mekelle, Ethiopia - a cross-sectional study. Adv Med Educ Pract. 2018;9:611-16.

73. Yu C, Liu Q, Wang W, Xie A, Liu J. Professional Identity of 0.24 Million Medical Students in China Before and During the COVID-19 Pandemic: Three Waves of National Cross-Sectional Studies. Front Public Health. 2022;10:868914.

74. El Hage S, Chahine MN, Sayde G, Daaboul M, El Masri J, Salameh P. Competencies required for graduated physicians: the integration of Englander's common taxonomy in a validated scale for the assessment of competency acquiring in undergraduate medical education. Ir J Med Sci. 2023;192(1):499-507.

75. Bußenius L, Harendza S, van den Bussche H, Selch S. Final-year medical students' self-assessment of facets of competence for beginning residents. BMC Med Educ. 2022;22(1):82.

76. Yamamoto T, Kawaguchi A, Otsuka Y. Developing the comprehensive medical professionalism assessment scale. MedEdPub. 2019;8(91):91.

77. Imm MR, Agarwal G, Zhang C, Deshpande AR, Issenberg B, Chandran L. EPMO: A novel medical student assessment tool that integrates entrustable professional activities, prime, and the modified Ottawa coactivity scale. Med Teach. 2023;45(4):419-25.

78. Yavari N, Asghari F, Shahvari Z, Nedjat S, Larijani B. Developing a comprehensive tool to assess professional attitude among physicians and medical students. J Med Ethics Hist Med. 2021;14:27.

79. Ramakrishna J, Valani R, Sriharan A, Scolnik D. Design and pilot implementation of an evaluation tool assessing professionalism, communication and collaboration during a unique global health elective. Med Confl Surviv. 2014;30(1):56-65.

80. Mostaghimi A, Olszewski AE, Bell SK, Roberts DH, Crotty BH. Erosion of Digital Professionalism During Medical Students' Core Clinical Clerkships. JMIR Med Educ. 2017;3(1):e9.

81. Jamalabadi Z, Ebrahimi S. Medical students' experiences and perspective on unprofessional behavior in clinical practice. J Adv Med Educ Prof. 2018;6(1):31-36.

82. Davis LE, King MK, Wayne SJ, Kalishman SG. Evaluating Medical Student Communication/Professionalism Skills from a Patient's Perspective. Front Neurol. 2012;3:98.

83. AlKhater SA. Perception of Saudi Undergraduate Students Towards Professionalism in Medicine. Sultan Qaboos Univ Med J. 2021;21(3):378-85.

84. Tabatabaei ZS, Mirzazadeh A, Amini H, Mafinejad MK. What we think about professional and unprofessional behaviors: differences between the perception of clinical faculty members and medical students. BMC Med Educ. 2022;22(1):866.

85. Jiang S, Xu Q, Su W, Chen X, Li Y, Tian Y, Xu K. Investigation on the occupation spirit evaluation and medical ethics construction of medical students. Lab Sci. 2015;18(3):223-27,30.

86. Shi Y. Investigation on the status quo of medical professionaliam of clinical interns and analysis of its influencing factors [Master]. JiLin Univ, 2019 (in Chinese).

87. Li Y, Chen X, Chen J, Liang L, Zhang G, Xie S, Zhang N, Tian Y. An empirical study of medical students' professionalism evaluation system. Med Philos. 2015;36(10):31-34 (in Chinese).

88. Liang J, Liu R, Lu Y, Sun L, Lai Y, Bai H, Liu Q, Mao Y. Research in professionalism of medical students at Shanghai Medical College of Fudan University. Chin J Med Educ. 2015;35(2):174-77,88 (in Chinese).

89. Zhang W, Chao L. Investigation on the current situation of professionalism of medical students in Baotou Medical College. J Baotou Med Col. 2021;37(5):119-22 (in Chinese).

90. Sun C, Zhang R, Luo Y. Effects of gratitude intervention on professionalism of medical students. Med Philos. 2018;39(1):49-51 (in Chinese).

91. Li R, Li Y, Peng J, Huang l, Zhang L, Zhang W, Zeng F, Qian Z, Lei G, Chen X, Tian Y. Comparative analysis and evaluation of residents’ and medical interns’ professionalism. Chin J Med Educ. 2016;36(6):905-10 (in Chinese).

92. Zhang S. Research on the status of professional identity and academic emotion of medical students [Master]. Fujian Normal Univ, 2009 (in Chinese).

93. Zhang L, Ma X. Investigation of medical students' professional identity and the countermeasures. PBIEdSch. 2016(21):72-74 (in Chinese).

94. Zhao X, Fang J, Jin S. Current situation and countermeasures of medical ethics education for intern doctors. J Bengbu Med Coll. 2009;34(5):450-51 (in Chinese).

95. Gao M. investication and analvsis of the current situation of medical ethics education of clinical medical students. J Fujian Med Univ (Soc Sci Ed). 2003;4(1):84-86 (in Chinese).

96. Cai X, Chen J, Wu X, Qu G, Sun J, Fang Y. Cognitive status of professionalism among medical students: a case study of two universities in China. Chin J Med Educ Res. 2020;19(3):359-63 (in Chinese).

97. Sun J, Wu X, Qu G, Cai X, Chen J, Fang Y. Study on physician's professionalism cognitive status among medical students. Chin J Med Educ. 2018;38:680-84 (in Chinese).

98. Yuan Y, Zeng Y, Ou Y, Zhang Y, Zheng X, Fang Y. Investigation of medical students' recognition of medical professionalism. Health Vocat Educ. 2022;40(1):111-12 (in Chinese).

99. Sun M, Liang L, Jiao M, Liu W, Tao S, Xue Y, Song W, Wan X, Zheng Y. Development and preliminary application of the measurement scale for medical students' professionalism cognition. Chin J Med Educ Res. 2021;20(1):86-90 (in Chinese).

100. Liu W, Liang L, Tao S, Kang Z, Gao L, Xu J, Song W, Xue Y. Analysis on the status of medical students' professional identity. Chin Higher Med Educ. 2019(3):6-8 (in Chinese).

101. Deng Y. Professionalism and Professional Identity among Medical Students in Three Colleges [Master]. Huazhong Univ Sci Technol, 2020 (in Chinese).

102. Fu Y, Shi S, Li H, Wang Z, Yue S, Zhang X, Zhang S, Jiang G. Investigation and research on different groups of people’s perception on medical professionalism in one 3A hospital in Beijing. Chin J Med Educ. 2016;36(3):376-80,424 (in Chinese).

103. Li Y, Fan G, Geng Q. Researches on the lndex System of Medical Students' Professionalism Evaluation Based on the Delphi and Comprehensive Evaluation Methods. Chin Health Serv Manag. 2020;37(6):467-71,80 (in Chinese).

104. Xu Y. Construction of tracking evaluation index system of clinical medicine graduates' training quality [Master]. Nanjing Med Univ, 2020 (in Chinese).

105. Chen M, Chen J, Wang L, Tang Y. Construction of the preliminary evaluation index system of post competencyof clinical undergraduates based on the standard of clinical medicine professional certification. J Med Res Combat Trauma Care. 2018;31(12):1305-08 (in Chinese).

106. Song W. The construction and preliminary application of milestone standards for cultivating medical professionalism in the "5+3" training model for clinical healthcare professionals [Master]. China Med Univ, 2020 (in Chinese).

107. Wang Y. Research on clinical medical students' professional identity based on salience hierarchy [Master]. HeNan Univ, 2015 (in Chinese).

108. Lv J. A study on the current situation and influencing factors of medical students' professionalism behavior in the social media environment - taking the fifth-grade medical students of clinical medicine major in a medical university as an example [Master]. China Med Univ, 2020 (in Chinese).

109. Zhang L. An investigation of professional identity of medical students [Master]. East China Normal Univ, 2010 (in Chinese).

110. Wu Y, Wang S, Ye X. The impact of professional commitment on professional identity of undergraduate medical students in the post-epidemic era. Chin Univ Stud Career Guide. 2022(6):56-64 (in Chinese).

111. Qiu S. The impact of local epidemic outbreaks on medical students' professional identity. Mod Bus Trade Ind. 2022;43(24):225-27 (in Chinese).

112. Hao J. The current situation and influencing factors of professional identity of undergraduate students majoring in dentistry in local medical colleges in Shanxi Province. Shanxi Med J. 2021;50(21):3004-06 (in Chinese).

113. Guo Z. lmpacts of Mortality Salience on Medical Students' Professional ldentity. J North Univ China Soc Sci Ed. 2018;34(6):98-103,09 (in Chinese).

114. Lv Y, Liu H, Yin X, Lv Y, Zhao R, Li Z, Gong S. Investigation and analysis on the professional identity status of medical students during the COVID-19 epidemic. Chin Med Ethics. 2020;33(8):954-58 (in Chinese).

115. Zhu K, Jiang S, Xu L, Zhao L, Tang B. Medical students' professional identity and related factors under the background of COVID-19 pandemic. Health Vocat Educ. 2021;39(10):57-59 (in Chinese).

116. Pan L, Yao W. Investigation of students' professional attitudes and professional identity at a provincial medical university during the COVID-19 pandemic. Health Vocat Educ. 2021;39(7):125-27 (in Chinese).

117. Cao X, Wang W, Sun Y. The influence of medical students' professional identity on academic emotions: the mediating role of self-efficacy. J Nanjing Univ Tradit Chin Med Soc Sci Ed. 2017;18(4):238-41 (in Chinese).

118. Wang F, Qu N, Niu Q. Professional identity among medical students: a cross-sectional analysis. Chin J Public Health. 2018;34(9):1260-62 (in Chinese).

119. Mei S. The relationship between medical students' professional identity, humanistic care ability, and career self-efficacy. J Wenzhou Med Univ. 2019;49(1):72-75,79 (in Chinese).

120. Yang Q, Kai W. Status quo and comparative study of professional identity of medical students in TCM hospitals. J Med Inform. 2018;31(10):116-18 (in Chinese).

121. Wu J. A comparative study of Chinese and American medical students' professional identity [Master]. Shanghai Int Stud Univ, 2014 (in Chinese).
